# The Prognostic Value of Platelet‐Albumin‐Bilirubin Score in Patients Undergoing Transcatheter Aortic Valve Replacement

**DOI:** 10.1002/clc.70100

**Published:** 2025-02-24

**Authors:** Xueting Duan, Liangzhen Qu, Han Chen

**Affiliations:** ^1^ Department of Cardiology The Third Affiliated Hospital of Zhejiang Chinese Medical University Hangzhou China; ^2^ Department of Cardiovascular Medicine International Institutes of Medicine, The Fourth Affiliated Hospital, Zhejiang University School of Medicine Yiwu China; ^3^ Department of Cardiology The Second Affiliated Hospital, Zhejiang University School of Medicine Hangzhou China

**Keywords:** all‐cause mortality, cardiovascular mortality, platelet‐albumin‐bilirubin score, transcatheter aortic valve replacement

## Abstract

**Background:**

Transcatheter aortic valve replacement (TAVR) has emerged as a well‐established treatment option for patients with aortic valve stenosis and/or regurgitation. However, risk stratification in patients indicated for TAVR remains challenging. This study aimed to evaluate the predictive value of the platelet‐albumin‐bilirubin (PALBI) score on post‐TAVR mortality.

**Methods:**

A total number of 812 patients with aortic stenosis and/or aortic regurgitation who underwent TAVR were consecutively enrolled in this study. PALBI score was calculated based on preoperative baseline bilirubin levels, albumin levels, and platelet counts. Patients were categorized into two groups according to the median PALBI score.

**Results:**

The median age of the study population at baseline was 74 (IQR: 69.00–80.00) years, and 58.6% were male. During the whole follow‐up period, all‐cause death was observed in 60 (7.4%) patients and 30 (3.7%) patients died due to cardiovascular events. According to multivariate analysis, a high PALBI score was independently associated with all‐cause mortality (HR = 2.679, 95% CI: 1.456–4.930, *p* = 0.002) and cardiovascular mortality (HR = 2.785, 95% CI: 1.133–6.849, *p* = 0.026). ROC curve analysis showed a significant predictive value of the PALBI score for all‐cause mortality (AUC = 0.633, 95% CI: 0.563–0.704, *p* = 0.001). Furthermore, the PALBI score strengthens the predictive value of the Society of Thoracic Surgeons (STS) score for all‐cause death after TAVR (STS score vs. PALBI score + STS score: AUC: 0.742 vs. 0.768).

**Conclusion:**

A high PALBI score was associated with increased all‐cause and cardiovascular mortality in patients after TAVR. PALBI score can further enhance the predictive potential of STS score for all‐cause mortality.

## Graphical abstract

A high platelet‐albumin‐bilirubin (PALBI) score was associated with increased all‐cause mortality in patients after transcatheter aortic valve replacement (TAVR). PALBI score can further enhance the predictive potential of the Society of Thoracic Surgeons (STS) score for all‐cause mortality.

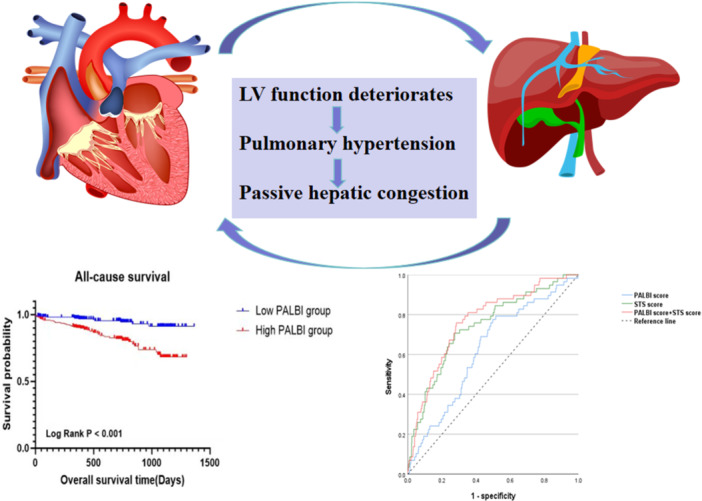

## 1 Introduction

Aortic valve disease is a common heart disease in the elderly population. The prevalence of aortic stenosis (AS) increases with age, averaging 0.2% at 50–59 years and increasing to 9.8% at 80–89 years [1]. Although aortic regurgitation (AR) is less common in the western population, it is more prevalent than AS in the Chinese population [2]. If no interventions were taken, the 2‐year mortality of severe AS is around 50% [3], and the mortality risk of AR patients is up to 20% per year [4]. Surgical aortic valve replacement (SAVR) has always been the primary treatment for patients with aortic valve disease [5]; however, a significant percentage of patients could not undergo surgery due to advanced age or other comorbidities. Since its first clinical application in 2002 [6], transcatheter aortic valve replacement (TAVR) has become an alternative treatment in patients with severe AS and a high perioperative risk [7]. Recently, the indications of TAVR have expanded to AS patients with intermediate and even selected low‐risk. Studies also demonstrate AR patients benefit from TAVR [8].

Although the TAVR devices and techniques keep improving, the 1‐year mortality rate remains high at 15%–20% [9]. The logistic European System for Cardiac Operative Risk Evaluation (EuroSCORE) and Society of Thoracic Surgeons (STS) score are widely used scoring systems for patients with valvular diseases. However, these scoring systems were initially designed to assess the risks of cardiac surgery, not specifically to assess TAVR risk [10, 11]. Moreover, chronic obstructive pulmonary disease (COPD), chronic kidney disease, atrial fibrillation, and frailty have also been identified as predictors of mortality after TAVR [12]. A practical scoring system with prognostic value after TAVR needs further research.

Liver dysfunction is common in patients with aortic valve disease. Previous studies have shown that bilirubin and albumin alone are associated with increased post‐TAVR mortality [13, 14]. The platelet‐albumin‐bilirubin (PALBI) score, based on measuring serum albumin and bilirubin levels and platelet counts, is an objective and integrated model to assess hepatic reserve function. It was initially proposed to predict the prognosis of liver cirrhosis. In subsequent studies, it was also found to be associated with the prognosis of hepatocellular carcinoma and other solid cancers [15, 16]. However, to the best of our knowledge, no relevant study has reported the association between the PALBI score and mortality after TAVR.

In the current study, a total number of 812 patients with AS and/or AR underwent TAVR were consecutively enrolled to evaluate the association between baseline PALBI score and post‐TAVR mortality.

## 2 Methods

### 2.1 Study Population

This study prospectively enrolled 924 consecutive patients aged > 18 years who were hospitalized for AS and/or AR who underwent TAVR in the Second Affiliated Hospital, Zhejiang University School of Medicine (SAHZU) from January 2018 to August 2022. Exclusion criteria were as follows: (1) unknown pre‐TAVR albumin levels, bilirubin levels, and/or platelet counts, (2) previous chronic liver cirrhosis, (3) chronic renal failure with or without peritoneal dialysis or hemodialysis, (4) patients were transferred to SAVR, (5) the follow‐up time was < 1 month. Finally, 812 patients who underwent successful TAVR procedures were included in our study. Written informed consent was obtained from all study subjects, and the study protocol was approved by the Human Research Ethics Committee at SAHZU.

Patients were followed up at 1 month and every year after the TAVR procedure. The last follow‐up visit for the current study occurred in October 2022. Follow‐up included regular office visits and phone calls to the patients or the next of kin.

### 2.2 Data Collection

Clinical characteristics, laboratory findings, and echocardiographic measurements of the participants were collected before TAVR. At baseline, the clinical characteristics included age, sex, body mass index (BMI), smoking status, STS score, New York Heart Association (NYHA) cardiac function class, medical history of dyslipidemia, diabetes mellitus, hypertension, stroke, myocardial infarction (MI), percutaneous coronary intervention (PCI), peripheral vascular disease (PVD), atrial fibrillation/flutter, COPD, and cancer.

Laboratory findings included platelet count, albumin, total bilirubin, hemoglobin, N‐terminal pro‐b‐type natriuretic peptide (pro‐BNP), creatinine, and troponin‐T (TnT) were measured from venous blood samples before TAVR procedures.

Echocardiography was performed by experienced cardiologists to record parameters including left ventricular ejection fraction (EF), left atrial diameter (LA), left ventricular end‐diastolic dimension (LVEDd), pulmonary arterial pressure grade, AR grade, and tricuspid regurgitation (TR) grade.

### 2.3 Assessment of PALBI Score Before TAVR

PALBI score was calculated as follows: 2.02 × log10 total bilirubin [µmol/L] − 0.37 × (log 10 bilirubin)^2^ − 0.04 × albumin [g/L] − 3.48 × log10 platelet count [1000/µL] + 1.01 × (log10 platelet count)^2^. The subjects were divided into two groups according to the median PALBI score on hospital admission: the low PALBI group (≦ −2.42, *n* = 402) and the high PALBI group (> −2.42, *n* = 410).

### 2.4 Study Endpoints

The primary study endpoint was all‐cause mortality after TAVR treatment. The secondary endpoint comprised cardiovascular mortality.

### 2.5 Statistical Analysis

SPSS Statistics for Windows (IBM, NY, USA), version 27.0, was used to perform all statistical analyses. Normally distributed data were reported as mean ± standard deviation and were compared using Student's *t*‐test, whereas non‐normally distributed data were presented as median and interquartile range (IQR) and were employed the Wilcoxon rank‐sum test to compare them. Categorical variables were expressed as numbers (percentage) and were compared using *χ* ^2^ tests or Fisher's exact test when appropriate. Kaplan–Meier analysis was used to evaluate and compare the time‐to‐event curve between the low and high PALBI score groups, with statistical significance examined using the log‐rank test. The Cox proportional hazard regression analysis was performed to clarify the association between the PALBI score and the incidence of death. For all regression analyses, only variables with a *p* < 0.05 in univariate analysis were incorporated into the multivariate model, with results reported as the hazard ratio (HR) and 95% confidence interval (CI). Receiver operating characteristic (ROC) curve analysis was used to evaluate the prognostic value of the PALBI score for all‐cause morality. A value of *p* < 0.05 was considered statistically significant.

## 3 Results

### 3.1 Study Population

A total number of 812 patients who underwent TAVR in SAHZU were included. The median age of the study population was 74.00 (IQR 69.00, 80.00) years, and 58.6% were male. The baseline clinical characteristics, laboratory findings, and echocardiographic measurements of the low and high groups are shown in Table 1.

**Table 1:** Baseline clinical characteristics, laboratory findings, echocardiographic measurements, and death events of the low and high PALBI groups.

	Low PALBI group (*n* = 402)	High PALBI group (*n* = 410)	*p* value
**Clinical characteristics**			
Age, yrs	73.00 (68.00, 79.00)	75.00 (70.00, 81.00)	< 0.001
Male	227 (56.5%)	249 (60.7%)	0.217
BMI, kg/m^2^	22.90 (20.35, 25.25)	22.35 (20.20, 24.80)	0.068
Smoker	102 (25.4%)	99 (24.1%)	0.685
STS score	3.14 (1.84, 5.57)	4.00 (2.25, 6.82)	0.002
NYHA class III/IV	302 (75.1%)	333 (81.2%)	0.035
Dyslipidemia	62 (15.4%)	44 (10.7%)	0.047
Diabetes mellitus	75 (18.7%)	68 (16.6%)	0.438
Hypertension	231 (57.5%)	224 (54.6%)	0.417
Prior MI	4 (1.0%)	6 (1.5%)	0.774
Prior PCI	39 (9.7%)	43 (10.5%)	0.710
Prior stroke	17 (4.2%)	30 (7.3%)	0.060
Atrial fibrillation/flutter	50 (12.4%)	101 (24.6%)	< 0.001
PVD	24 (6.0%)	31 (7.6%)	0.367
COPD	75 (18.7%)	104 (25.4%)	0.021
Cancer	18 (4.5%)	26 (6.3%)	0.241
Type of AVD			0.041
PAS	220 (54.7%)	189 (46.1%)	
PAR	72 (17.9%)	94 (22.9%)	
AS + AR	110 (25.4%)	127 (31.0%)	
**Laboratory findings**			
Hemoglobin, g/L	126.00 (115.00, 137.00)	127.00 (115.00, 140.00)	0.445
Pro‐BNP, pg/mL	982.00 (307.00, 2522.50)	2133.00 (666.25, 6384.00)	< 0.001
Creatinine, μmol/L	75.00 (60.15, 92.00)	77.00 (64.38, 98.00)	0.053
Tn‐T, ng/mL	0.018 (0.012, 0.030)	0.029 (0.016, 0.054)	< 0.001
**Echocardiographic measurements**			
EF, %	60.80 (53.70, 65.85)	56.30 (42.90, 63.10)	< 0.001
LA, cm	4.07 (3.66, 4.46)	4.32 (3.89, 4.77)	< 0.001
LVEDd, cm	4.84 (4.32, 5.55)	5.25 (4.53, 5.91)	< 0.001
PAH			< 0.001
None (0)	311 (77.4%)	251 (61.2%)	
Mild (1)	54 (13.4%)	74 (18.0%)	
Moderate (2)	27 (6.7%)	58 (14.1%)	
Severe (3)	10 (2.5%)	27 (6.6%)	
MR			< 0.001
None (0)	86 (21.4%)	50 (12.2%)	
Mild (1)	212 (52.7%)	199 (48.5%)	
Mild to moderate (2)	48 (11.9%)	45 (11.0%)	
Moderate (3)	39 (9.7%)	83 (20.2%)	
Moderate to severe (4)	10 (2.5%)	17 (4.1%)	
Severe (5)	7 (1.7%)	16 (3.9%)	
TR			< 0.001
None (0)	136 (33.8%)	114 (27.8%)	
Mild (1)	222 (55.2%)	195 (47.6%)	
Mild to moderate (2)	16 (4.0%)	36 (8.8%)	
Moderate (3)	20 (5.0%)	38 (9.3%)	
Moderate to severe (4)	2 (0.5%)	9 (2.2%)	
Severe (5)	6 (1.5%)	18 (4.4%)	
**Death events**			
1‐month			
All‐cause	5 (1.2%)	7 (1.7%)	0.584
Cardiovascular	4 (1.0%)	7 (1.7%)	0.380
1‐year			
All‐cause	9 (2.2%)	27 (6.6%)	0.003
Cardiovascular	5 (1.2%)	15 (3.7%)	0.026
Whole follow‐up			
All‐cause	15 (3.7%)	45 (11.0%)	< 0.001
Cardiovascular	7 (1.7%)	23 (5.6%)	0.003

*Note:* Dyslipidemia was defined as low‐density lipoprotein cholesterol levels ≥ 160 mg/dL, high‐density lipoprotein cholesterol levels < 40 mg/dL, triglycerides levels ≥ 150 mg/dL, or lipid‐lowering medication use. Diabetes mellitus was defined as fasting blood glucose levels ≥ 126 mg/dL or antidiabetic medication use. Hypertension was defined as systolic blood pressure ≥ 140 mmHg, diastolic blood pressure ≥ 90 mmHg, or antihypertensive medication use. Based on the pulmonary systolic blood pressure (PASP) measured by Doppler echocardiography, the severity of pulmonary arterial hypertension can be classified into mild (35–49 mmHg), moderate (50–69 mmHg), and severe (≥ 70 mmHg). MR/TR: 0—none, 1—mild, 2—mild to moderate, 3—moderate, 4—moderate to severe, 5—severe. PAH: 0—none, 1—mild, 2—moderate, 3—severe.

Abbreviations: BMI, body mass index; STS, Society of Thoracic Surgeons; NYHA, New York Heart Association; MI, myocardial infarction; PCI, prior percutaneous coronary intervention; PVD, peripheral vascular disease; COPD, chronic obstructive pulmonary disease; PAS, pure aortic stenosis; PAR, pure aortic regurgitation; Pro‐BNP, pro‐B‐type natriuretic peptide; TnT, troponin T; EF, ejection fraction; LA, left atrium; LVEDd, left ventricular end‐diastolic dimension; PAH, pulmonary arterial hypertension; MR, mitral regurgitation; TR, tricuspid regurgitation.

Participants in the high PALBI group were older and had higher STS scores, higher pro‐BNP levels, higher Tn‐T levels, and worse NYHA class. They also had a higher prevalence of atrial fibrillation/flutter and COPD.

Regarding echocardiographic features, lower EF, larger left atrial diameters, and larger left ventricular dimensions were observed in the high PALBI group (*p* < 0.05). A high PALBI score was also associated with more severe concomitant MR and TR, as well as a higher proportion of moderate/severe pulmonary arterial hypertension.

No significant disparities were found in sex, BMI, smoking status, hemoglobin, and creatinine. In addition, there was no association between PALBI score and comorbidities of patients undergoing TAVR, including diabetes mellitus, hypertension, stroke, PCI, MI, stroke, PVD, and cancer (*p* > 0.05 for all).

### 3.2 PALBI Score and Death Events

During the 1‐month to 3‐year follow‐up period, 60 (7.4%) patients suffered from all‐cause death, and 30 (3.7%) patients died due to cardiovascular events (Table 1). Compared with the low PALBI group, the all‐cause mortality (11.0% vs*.* 3.7%, *p* < 0.001) and the cardiovascular mortality (5.6% vs*.* 1.7%, *p* = 0.003) at the whole follow‐up were significantly higher in patients with high PALBI scores. Similarly, the higher all‐cause and cardiovascular mortality at the 1‐year follow‐up were also found in the high PALBI group. However, no significant difference in all‐cause or cardiovascular mortality at the 1‐month follow‐up was noted between the low and high PALBI groups.

**Figure 1:**
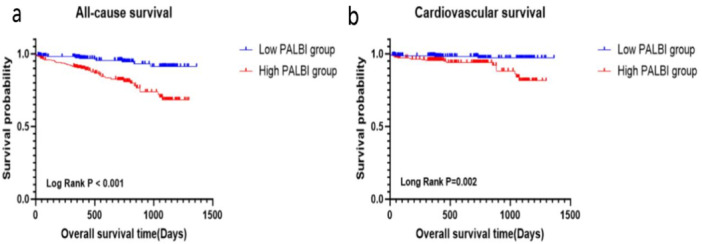
Kaplan–Meier curves for all‐cause death (a) and cardiovascular death (b) at the whole follow‐up of patients with different PALBI scores.

To compare the time‐to‐event curve between the different PALBI score groups, Kaplan–Meier survival analysis with the log‐rank test of all‐cause and cardiovascular mortality was performed among all patients. The results indicated a significantly higher incidence of all‐cause death in the high PALBI group throughout the entire follow‐up in comparison with the low PALBI group (log‐rank *p* < 0.001) (Figure 1a). Similarly, cardiovascular mortality exhibited significant differences between the two PALBI score groups (log‐rank *p* = 0.002) (Figure 1b).

To confirm the association between PALBI score and all‐cause mortality in patients who underwent TAVR, we conducted a Cox regression analysis (Table 2). Univariate Cox regression analysis indicated that significantly increased risk of all‐cause mortality at the whole follow‐up for patients with higher PALBI scores (HR = 3.287, 95% CI 1.832–5.901, *p* < 0.001). After adjusting for age, NYHA class, STS score, PVD, atrial fibrillation/flutter, hemoglobin, creatinine, Tn‐T, left atrium size, mitral regurgitation, and TR in multivariate Cox regression analyses, a higher PALBI score was still associated with increased risk of all‐cause death (HR = 2.679, 95% CI 1.456–4.930, *p* = 0.002).

**Table 2:** Cox regression analysis for determining predictors of all‐cause mortality.

	Univariate HR [95% CI]	*p* value	Multivariate HR [95% CI]	*p* value
Age	1.085 (1.045–1.125)	< 0.001		
STS score	1.070 (1.048–1.092)	< 0.001		
NYHA class	3.631 (1.316–10.024)	0.013		
PVD	3.149 (1.672–5.931)	< 0.001	2.014 (1.010–4.015)	0.047
Atrial fibrillation/flutter	1.844 (1.039–3.273)	0.036		
Hemoglobin	0.975 (0.963–0.986)	< 0.001	0.986 (0.973–0.999)	0.035
Creatinine	1.009 (1.005–1.013)	< 0.001		
TnT	1.076 (1.043–1.109)	< 0.001	1.050 (1.014–1.087)	0.008
LA	1.475 (1.038–2.097)	0.030		
MR	1.272 (1.053–1.538)	0.013		
TR	1.311 (1.083–1.587)	0.005		
PALBI score	3.287 (1.832–5.901)	< 0.001	2.679 (1.456–4.930)	0.002

We next investigated the association between PALBI score and cardiovascular death (Table 3). Univariate analysis revealed that higher age, lower STS score, higher prevalence of NYHA class, lower hemoglobin levels, higher creatinine, higher TnT, higher proportion of MR and AR, and higher PALBI score were associated with cardiovascular mortality (*p* < 0.05). Multivariate analysis showed that lower hemoglobin levels (HR = 0.979, *p* = 0.029), higher creatinine (HR = 1.009, *p* = 0.009), higher Tn‐T (HR = 1.054, *p* = 0.004), and higher PALBI score (HR = 2.785, *p* = 0.026) remained as independent predictors for cardiovascular mortality.

**Table 3:** Cox regression analysis for determining predictors of cardiovascular mortality.

	Univariate HR [95% CI]	*p* value	Multivariate HR [95% CI]	*p* value
Age	1.075 (1.021–1.132)	0.006		
STS score	1.060 (1.028–1.093)	< 0.001		
NYHA class	2.583 (1.431–4.662)	0.002		
Hemoglobin	0.966 (0.950–0.982)	< 0.001	0.979 (0.961–0.998)	0.029
Creatinine	1.011 (1.006–1.015)	< 0.001	1.009 (1.002–1.016)	0.009
TnT	1.076 (1.044–1.110)	< 0.001	1.054 (1.017–1.093)	0.004
MR	1.349 (1.042–1.745)	0.023		
TR	1.320 (1.017–1.713)	0.037		
PALBI score	3.533 (1.514–8.241)	0.003	2.785 (1.133–6.849)	0.026

**Figure 2:**
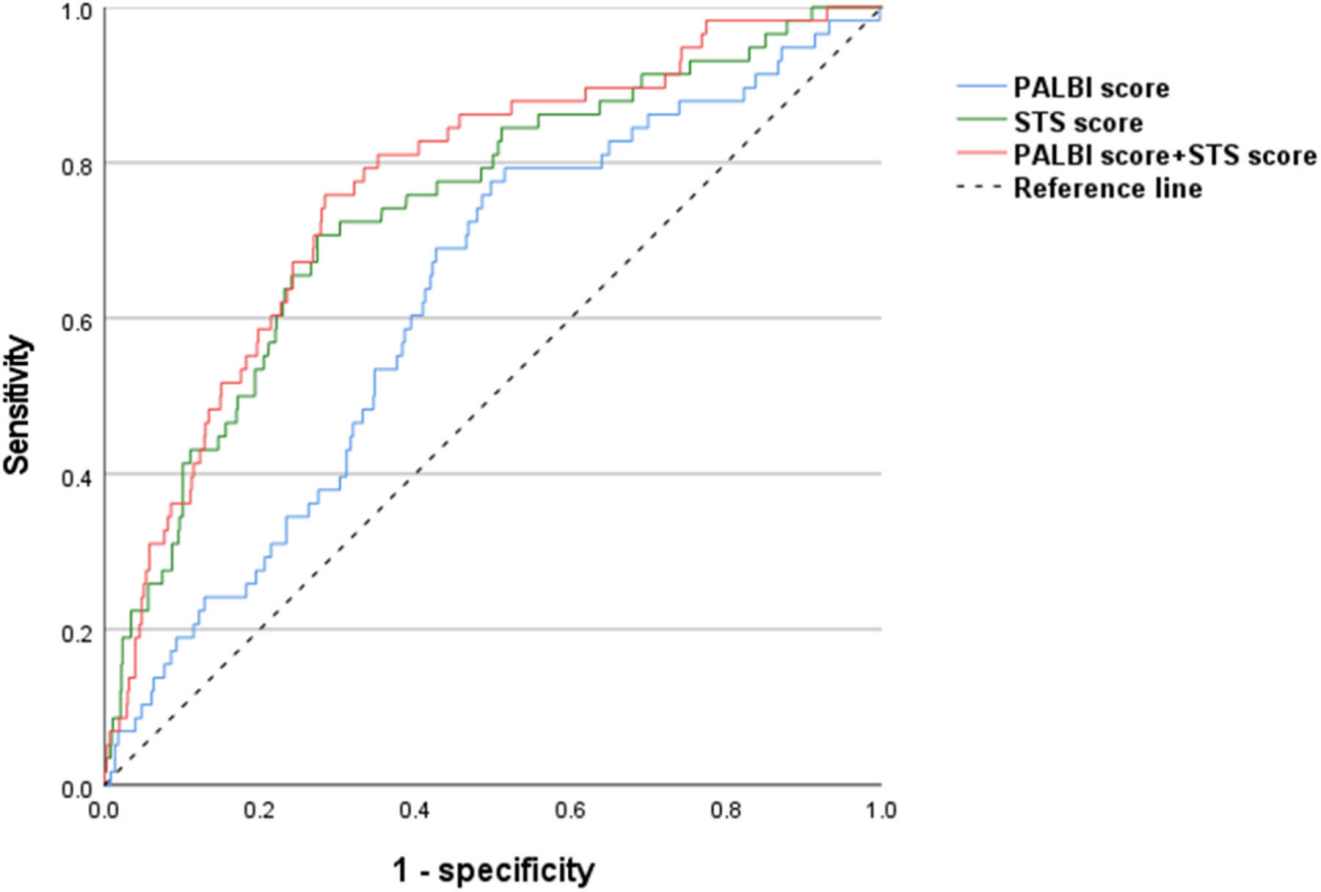
ROC curve of PALBI score, STS score, and the combination of PALBI score and STS score for detecting all‐cause mortality.

Further, ROC curve analysis showed a significant predictive value of the PALBI score for all‐cause mortality at the whole follow‐up (AUC = 0.633, 95% CI 0.563–0.704, *p* = 0.001) (Figure 2). Moreover, the PALBI score could strengthen the predictive value of the STS score for all‐cause death after TAVR (STS score vs. PALBI score + STS score: AUC: 0.742 vs. 0.768).

## 4 Discussion

This study analyzed the association between PALBI score and post‐mortality in patients undergoing TAVR. We found that: (1) the PALBI score is an independent prognosticator of all‐cause and cardiovascular death; (2) the PALBI score can strengthen the predictive value of the STS score model in patients who underwent TAVR.

Prior studies have evaluated the prognostic clinical significance of different liver function tests in patients undergoing TAVR. Stephanie and colleagues pointed out that pre‐TAVR hyperbilirubinemia (≧ 1.2 mg/dL) was an independent predictor of post‐TAVR in‐hospital mortality and stroke [13]. Andrada and colleagues showed that low preprocedural albumin (≤ 4 g/dL) was independently associated with a 2.28‐fold increase in 2.1‐year all‐cause mortality [17]. Similarly, another study used 3.5 g/dL as a cut‐off value and found that patients with low albumin concentrations had prolonged intensive care unit (ICU) length of stay and a lower possibility of surviving to 90 days [18]. Stolz and colleagues demonstrated that cardiohepatic syndrome, which was defined as an elevation of at least two of three laboratory cholestasis parameters of hepatic cholestasis (bilirubin, alkaline phosphatase, and gamma‐glutamyl transferase), was correlated with more severe heart failure symptoms at baseline and follow‐up, as well as lower 3‐year survival rates [19]. The severe thrombocytopenia after TAVR was independently associated with both early‐ and long‐term mortality [20], however, the correlation between baseline decreased platelet counts and mortality after TAVR is unknown. In recent years, the joint scoring system has been widely used to judge the liver function reserve of patients, including the Child‐Pugh classification (CP), Model of End‐Stage Liver Disease (MELD) score, and PALBI score. The CP has some disadvantages, including subjective factors (ascites and encephalopathy) and interrelated factors (serum albumin and ascites), and it was not established statistically [21]. MELD score is calculated using creatinine, total bilirubin, and international normalized ratio (INR), whereas INR may elevate due to anticoagulation and vary among laboratories [22]. In addition, MELD provided limited risk information in patients treated with oral anticoagulation [22]. Therefore, the PALBI score, as a purely objective score based on albumin, total bilirubin, and platelet counts, may become a more practical prognostic index in patients undergoing TAVR.

Liver congestion might be a bridge between liver function and aortic valve disease. In the context of long‐standing aortic valve disease, LV function deteriorates, and cardiac remodeling occurs [23]. Then, patients might suffer from subsequent MR, increasing LA pressures, and lead to pulmonary hypertension. When the right ventricular (RV) is unable to respond to an excessively increasing afterload, RV dilates, RV function decreases, and secondary TR could be observed. Ultimately, the abovementioned process leads to chronic systemic venous congestion with corresponding end‐organ damage, including passive hepatic congestion [24]. On the other hand, diminished cardiac output will also result in impaired liver perfusion.

Passive congestion and impaired perfusion will induce low hepatic blood flow and hypoxic hepatocellular injury, then causing impaired uptake of indirect bilirubin from the blood, eventually leading to an increase in serum bilirubin level [25]. Regarding albumin, it is possible that microvascular protein escape increases, and intestinal congestion favors albumin enteric losses when systemic congestion [26, 27]. Decreased platelet counts are caused by hypersplenism due to passive liver congestion, suggesting the severity of portal hypertension [13].

During the TAVR procedure, balloon dilation and valve implantation may make the aortic valve almost entirely occlusive, resulting in interrupted blood flow and a sharp rise in intra‐aortic pressure. The latter leads to increased left ventricular pressure, mitral regurgitation, and pulmonary hypertension. The above mentioned process may lead to increased central venous pressure and hepatic congestion. Additionally, hemodynamic changes in the setting of tissue injury during aortic valve replacement may play an essential part in the activation and aggregation of circulating platelets with subsequent removal from circulation, finally leading to platelet count decreases [28].

It is well‐known that levels of bilirubin and albumin are linked with central venous pressure in patients with heart failure [29, 30]. Therefore, it can be postulated that liver congestion resulting from right‐sided heart overload is characterized by the PALBI score. In the current study, patients with a high PALBI score presented with higher rates of concomitant MR, moderate/severe pulmonary arterial hypertension, and TR when compared with those with a low PALBI score, suggesting that a high PALBI score is associated with right‐sided heart overload in aortic valve disease patients. Furthermore, patients with a high PALBI score had higher pro‐BNP and TnT, as well as a higher prevalence of atrial fibrillation/flutter, which generally contributes to the incidence of all‐cause deaths after TAVR [12, 31].

Multiple clinical trials have reported the beneficial effects of TAVR on patients with pure AR [32]. However, most prior clinical trials exclude AR patients and do not provide prognostic information for these patients. Our study demonstrated the association between PALBI score and post‐TAVR mortality in patients with AS and/or AR. Although the predictive value of PALBI score for mortality is moderate (AUC = 0.633), other scoring systems that are currently used for risk stratification in patients undergoing TAVR, such as EuroSCORE, EuroSCORE II, Society of Thoracic Surgeons predicted risk of mortality (STS‐PROM) and German aortic valve (GAV) score, were also relatively modest in discriminatory power (maximum AUC of 0.711, minimum AUC of 0.661 have been reported) and could not provide a thorough risk evaluation before TAVR [33]. Moreover, the PALBI score is relatively simple and objective compared to existing scoring systems, which are calculated using three common clinical laboratory indicators and do not require additional testing costs. Furthermore, current guidelines and established risk scores do not incorporate liver function in decision‐making and risk assessment [34]. The impact of liver function is not included in EuroScore, while the STS score only mentions the presence of liver disease without further definition.

In patients undergoing TAVR, the high residual mortality emphasizes the need for more strategic patient selection. Our study identifies the PALBI score, in addition to the STS score, as valuable tools for risk stratification in TAVR. The combination of the PALBI score with the STS score allowed the identification of patient subgroups with vastly differing outcomes after TAVR. Further investigation should explore how these two risk scores can be integrated into the decision‐making process in patients considered for TAVR, especially which patient should undergo TAVR and which should not. Additionally, to provide a thorough risk evaluation before TAVR, it is important to emphasize that risk evaluation should be carried out for each case by a multidisciplinary team.

The current study has several limitations. First, the study was restricted to a single center with limited patients. Second, we did not calculate the PALBI score after TAVR. Therefore, whether the dynamic changes in the PALBI score were associated with clinical outcomes after TAVR remains unknown. Our findings need to be validated in a large‐scale, multicenter prospective study.

## 5 Conclusion

In patients undergoing TAVR, a high PALBI score of > −2.42 was an independent prognosticator of all‐cause and cardiovascular death. The predictive significance of the STS score + PALBI score was superior to the STS score. These findings indicate that the PALBI score might be a promising index for identifying appropriate individuals for the TVAR procedure.

## Conflicts of Interest

The authors declare no conflicts of interest.

## Data Availability

The data sets generated and/or analyzed during the current study are available from the corresponding author on reasonable request.
